# Draft Genome Sequence of Bacillus coagulans MA-13, a Thermophilic Lactic Acid Producer from Lignocellulose

**DOI:** 10.1128/MRA.00341-19

**Published:** 2019-06-06

**Authors:** Martina Aulitto, Salvatore Fusco, Carl Johan Franzén, Andrea Strazzulli, Marco Moracci, Simonetta Bartolucci, Patrizia Contursi

**Affiliations:** a Department of Biology, University of Naples Federico II, Naples, Italy; b Division of Industrial Biotechnology, Department of Biology and Biological Engineering, Chalmers University of Technology, Gothenburg, Sweden; c Task Force on Microbiome Studies, University of Naples Federico II, Naples, Italy; University of Arizona

## Abstract

Bacillus coagulans MA-13 is an efficient lactic acid producer which withstands high concentrations of the growth inhibitors formed during the pretreatment of lignocellulosic feedstock. This draft genome sequence is expected to pave the way toward the understanding of mechanisms responsible for the robustness of MA-13 during simultaneous saccharification and fermentation.

## ANNOUNCEMENT

Bacillus coagulans MA-13 is a Gram-positive spore-forming moderately thermophilic facultatively anaerobic bacterium isolated from processed bean waste (1). MA-13 ferments lignocellulose-derived hexoses to lactic acid (LA); therefore, it is a suitable candidate for the conversion of lignocellulose to LA, which is a building block for the production of polylactic acid (PLA), i.e., a biodegradable bioplastic (2). Recently, MA-13 was used for the conversion of steam-exploded wheat straw to LA in simultaneous saccharification and fermentation (SSF) (3, 4). The preexposure to the inhibitor-rich lignocellulosic hydrolysate (5) led to a physiological adaptation of MA-13, which was reflected in an improved fermentation performance during SSF, thus resulting in a more cost-effective process (4).

The strain MA-13 was isolated and cultivated as previously described (1) before genomic DNA was extracted using the LETS (lithium, EDTA, Tris, and SDS) buffer method, followed by phenol extraction (6). The sequencing of the whole genome was performed using the Illumina NextSeq platform at Genomix4life S.R.L. (Salerno, Italy) with paired-end indexed libraries prepared using a Nextera XT kit (Illumina, Inc.). The reads (151 nucleotides [nt]) were *de novo* assembled using the SPAdes genome assembler version 3.9.0 on BaseSpace (7, 8). A total of 11,245,275 paired-end reads with an average length of 150 base pairs (bp) were assembled into 1,653 contigs (*N*_50_ length of 51,225 nt, *N*_90_ length of 4,278 nt), with the largest contig being 145,076 nt long. The draft genome consists of 3,237,270 bp with a GC content of 47.11%.

Functional annotation of contigs was carried out using the comprehensive bioinformatics tool Blast2GO version 5.2.5 (9, 10). A total of 3,336 open reading frames (ORFs) were identified, 3,268 of which were predicted as genes. A further annotation analysis was carried out with Rapid Annotations using Subsystems Technology (RAST) software (myRAST version 36) (11). Default parameters were used for all software unless otherwise specified. There were 2,355 gene ontology (GO) terms, 468 of which were assigned to the category of biological processes, including all necessary genes for the glycolysis (Embden-Meyerhof-Parnas) and the tricarboxylic acid cycle. Moreover, one d-lactate and three l-lactate dehydrogenase genes were identified, which can account for the superior fermentation performance of MA-13 (1, 4). The tolerance toward lignocellulose-derived inhibitors can be traced back to the presence in the MA-13 genome of genes encoding enzymes putatively involved in detoxification pathways, i.e., four aldehyde dehydrogenases, three short-chain dehydrogenases, two alcohol dehydrogenases, and one zinc-dependent alcohol dehydrogenase, which are potentially associated with detoxification reactions (12–14). Besides LA metabolism, MA-13 possesses genes required for the production of value-added chemicals, such as acetoin, butanediol, and polyhydroxybutyrate (i.e., a biodegradable plastic). The presence of genes encoding bacteriocins is related to the production of antimicrobial molecules (15–17) suitable to avoid competition with other bacteria in nonsterile open fermentation. As shown for other *B. coagulans* strains (18–21), genes associated with the defense mechanism toward foreign genetic elements, i.e., the clusters of regularly interspaced short palindromic repeat (CRISPR)-cas systems (22), were identified using CRISPRFinder version 1.3 (23).

### Data availability.

This whole-genome shotgun project has been deposited at DDBJ/ENA/GenBank under the accession number SMSP00000000. The version described in this paper is version SMSP01000000. The raw reads have been deposited in the SRA under the accession number PRJNA526660 and are also available at https://www.ncbi.nlm.nih.gov/Traces/study/?acc=PRJNA526660.
